# Highly Doped Carbon Nanotubes with Gold Nanoparticles and Their Influence on Electrical Conductivity and Thermopower of Nanocomposites

**DOI:** 10.1371/journal.pone.0044977

**Published:** 2012-09-14

**Authors:** Kyungwho Choi, Choongho Yu

**Affiliations:** Department of Mechanical Engineering, Texas A&M University, College Station, Texas, United States of America; University of California, Merced, United States of America

## Abstract

Carbon nanotubes (CNTs) are often used as conductive fillers in composite materials, but electrical conductivity is limited by the maximum filler concentration that is necessary to maintain composite structures. This paper presents further improvement in electrical conductivity by precipitating gold nanoparticles onto CNTs. In our composites, the concentrations of CNTs and poly (vinyl acetate) were respectively 60 and 10 vol%. Four different gold concentrations, 0, 10, 15, or 20 vol% were used to compare the influence of the gold precipitation on electrical conductivity and thermopower of the composites. The remaining portion was occupied by poly(3,4-ethylenedioxythiophene) poly(styrenesulfonate), which de-bundled and stabilized CNTs in water during synthesis processes. The concentrations of gold nanoparticles are below the percolation threshold of similar composites. However, with 15-vol% gold, the electrical conductivity of our composites was as high as ∼6×10^5^ S/m, which is at least ∼500% higher than those of similar composites as well as orders of magnitude higher than those of other polymer composites containing CNTs and gold particles. According to our analysis with a variable range hopping model, the high conductivity can be attributed to gold doping on CNT networks. Additionally, the electrical properties of composites made of different types of CNTs were also compared.

## Introduction

Carbon nanotubes (CNTs) have been considered as promising candidates for various applications including field effect transistors (FETs) [1], [2], touch screens [3], [4], field emission displays (FEDs) [5], [6], and solar cells [7]–[9] due to their outstanding electrical properties. Recently, CNTs were used as fillers in polymer composites and their electrical conductivities were orders of magnitude higher than other polymer composites with conductive fillers [10]–[14]. It has been shown that the electrical conductivity can be dramatically increased as a function of nanotube loadings in the composites. The highest electrical conductivity was obtained with 60 wt%, but the conductivity was decreased with composites containing CNTs more than 60 wt% [14]. The reduction in electrical conductivity is due to CNT aggregations caused by the insufficient amount of dispersants (which cannot be increased due to high CNT loadings). The optimum ratio of CNT to stabilizer for high electrical conductivity was found to be 3∶2. This means that the maximum CNT concentration should not be larger than 60 wt% for improving conductivity.

In this work, we demonstrate that nanoparticles can be incorporated on nanotube surfaces in order to further improve the electrical conductivity of nanocomposites. This also provides the influence of spherical-shape metal nanoparticles on the electrical conductivity of polymer composites. Nanoparticles can be precipitated on nanotubes by galvanic displacement or reduction potential differences between nanotubes and nanoparticles [15]. When nanoparticles are precipitated on nanotubes, charge transfer between them occurs, altering electrical transport properties of the nanotubes. Such property changes are similar to semiconductor doping with an acceptor impurity. For convenience, we shall therefore refer to the nanoparticle precipitation process as ‘doping’. In this paper, we particularly studied the influence of gold nanoparticle incorporation into CNT-filled composites on their electrical properties.

The electrical properties were measured with three different gold concentrations, 10, 15, or 20 vol%, in order to identify the effect of p-type doping on the conductivity, dispersion, and microstructure of the resulting composites. The maximum electrical conductivity was measured to be ∼6×10^5^ S/m with 60 vol% of single wall carbon nanotubes (SWCNTs) and 15 vol% of gold nanoparticles. This electrical conductivity is orders of magnitude higher than those of other polymer composites with comparable concentration of gold nanoparticle (10^−4^∼10^2^ S/m) [16]–[18]. With the variable range hopping model, the effect of p-type doping was also analyzed. Furthermore, composites containing different type CNTs were also synthesized, and their electrical properties and microstructures were presented in the following sections.

## Results and Discussion

**Figure 1:**
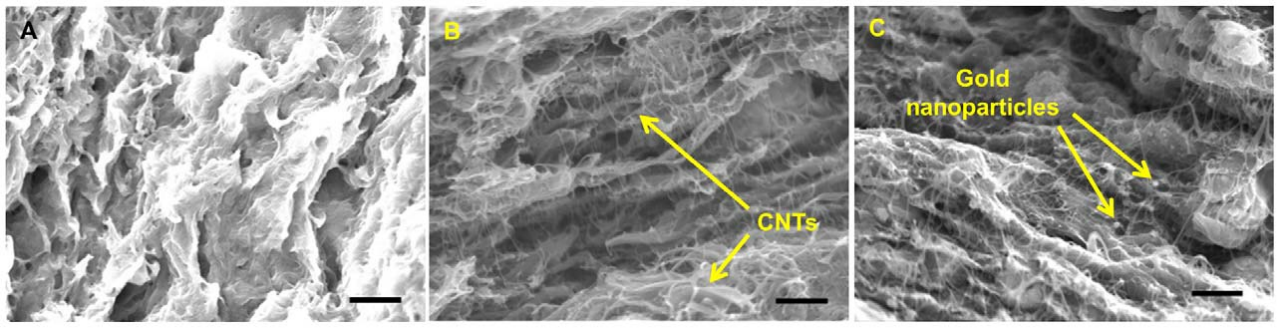
Cold-fractured cross sections of Sample 4 (A), Sample 5 (B), Sample 6 (C) (see **Table 1**). With increasing gold vol% and decreasing PEDOT∶PSS vol%, more CNTs were pulled out from the surface. The arrows indicate CNTs and gold nanoparticles. All scale bars indicate 1 µm.

All samples contain 60-vol% CNTs and 10-vol% PVAc, and the rest 30 vol% was PEDOT∶PSS or PEDOT∶PSS with gold (2∶1, 1∶1, and 1∶2 ratios), as listed in Table 1. For the samples containing SWCNT (Sample #: 1∼6), many nanotubes in the sample with 20-vol% PEDOT∶PSS were embedded (Figure 1A) whereas the samples with 15- and 10-vol% PEDOT∶PSS show more nanotubes separated from the polymer (Figure 1B and 1C), presumably due to less stabilizers. Gold nanoparticles were observed in the sample with 20-vol% gold (Figure 1C). Two PVAc polymer with different *T*_g_ (Vinnapas 401 and 600BP) were used, but we did not find any noticeable differences in microstructures. The films made from Vinnapas 600BP were more flexible than those made from Vinnapas 401 at room temperature due to the higher *T_g_* of Vinnapas 401 than that of 600BP.

**Table 1:** List of the composites with all contents and their vol%.

Sample number	CNT type	CNT vol%	PEDOT∶PSS vol%	Au vol%	PVAc vol%		Drying time (hr) at 80°C
					401	600BP	
1	HSWCNT	60	20	10	10	-	2
2	HSWCNT	60	15	15	10	-	2
3	HSWCNT	60	10	20	10	-	2
4	HSWCNT	60	20	10	-	10	6
5	HSWCNT	60	15	15	-	10	6
6	HSWCNT	60	10	20	-	10	6
7	MWCNT	60	30	-	-	10	6
8	CSWCNT	60	30	-	-	10	6
9	MWCNT	60	15	15	-	10	6
10	CSWCNT	60	15	15	-	10	6

Three different CNT type and two different PVAc were used with varying gold nanoparticle concentrations. The samples were synthesized by drying aqueous mixtures at room temperature for 48 hrs and subsequently at 80°C for 2 or 6 hrs.

**Figure 2:**
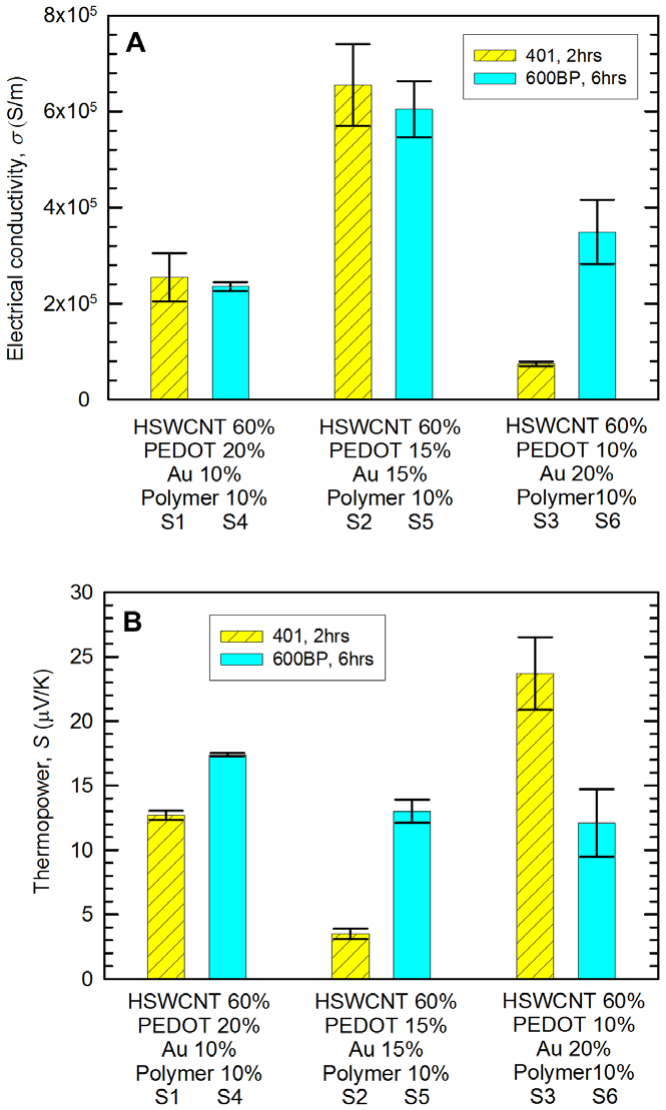
Electrical conductivity (A) and thermopower (B) of Sample 1∼6 (see **Table 1**). The vol% of HSWCNT and polymer (Vinnapas 401 or BP600) was 60 and 10. The ratio of PEDOT∶PSS to gold was 2∶1, 1∶1, or 1∶2.

Figure 2A shows the electrical properties of Sample 1∼6. The electrical conductivity was increased when the gold content was increased from 10 (Sample 1 and 4) to 15 vol% (Sample 2 and 5). The highest electrical conductivity was measured to be ∼6×10^5^ S/m with 15-vol% PEDOT∶PSS, 15-vol% gold, and 60-vol% SWCNT. This value is orders of magnitude higher than those of other nanotube-filled polymer composites [10], [11] and shows ∼500% improvement compared to our previous work with similar amounts of SWCNT and PH1000 (∼9×10^4^ S/m) [14]. It is likely that the electrical conductivity of gold is not the only reason that we obtained such high electrical conductivity from the composites. This is because the typical percolation threshold of gold nanoparticles is ∼30 vol% in polymer composites [18], which is larger than the maximum gold concentration (20 vol%) in our experiments. In other words, when the concentration of the nanoparticles is lower than the percolation threshold, the mean distance between the nanoparticles is too large to have connected gold networks. For example, Devasdoss et al. showed that the maximum electrical conductivity is 8×10^−8^ S/m with a composite containing gold nanoparticles (mole ratio of 4.95×10^−2^) and metallopolymer [16]. Podhaecka et al. reported that the electrical conductivity of a composite with gold nanoparticles (∼10 vol%) and poly(3-octylthiophene) is 10^−4^ S/m [17]. A high gold nanoparticle concentration, 40 vol% well above the percolation threshold in poly-4-vinyl pyridine matrices resulted in only ∼10^2^ S/m [18]. Such lower electrical conductivities suggest the high electrical conductivity from our samples is likely from p-type doping on nanotubes by the nanoparticles.

Gold nanoparticles are easily precipitated by spontaneous reduction [9], [15], [19] due to the larger reduction potential of gold ions ([AuCl_4_]^−^+3e^−^→Au(s)+4Cl^−^, standard electrode potential (*E* ^0^) = +0.93∼1.002 V) [15], [20]–[22] than those of nanotubes [15]. This causes nanotubes to donate electrons to gold, thereby increasing hole carrier concentrations [23], [24]. The work functions of SWCNTs (4.5∼5.0 eV) [25], [26] and MWCNTs (4.3∼4.95 eV) [25], [27] are also smaller than that of gold (5.1∼5.47 eV [28], [29]), making electrons transferred from nanotubes to gold nanoparticles. The electrical conductivity of the sample with 20-vol% gold (sample 3 and 6) is lower than those of the samples containing 15-vol% gold. The inferior conductivity with the higher gold concentration also suggests that gold nanoparticles themselves did not make percolated paths or significantly affect the electrical conductivity of our composites. We believe this is due to the poor nanotube dispersion caused by the small volume fraction of PEDOT∶PSS, which de-bundles and disperses carbon nanotubes in water. When large carbon nanotube bundles are present in the composites, the number of tube-tube junctions decreases, resulting in electrically more resistive nanotube networks [14], [30]. Furthermore, the composite contains more pores because large bundles are not readily embedded in the polymer due to increased stiffness.

Two different annealing conditions (2 hrs and 6 hrs at 80°C) were tested to identify any changes in electrical properties. The longer annealing time made the sample mechanically stronger but the electrical conductivities of the samples containing 10- or 15-vol% gold are not strongly dependent on the drying condition. When the gold concentration was increased to 20 vol% (S3 and S6), the longer drying time resulted in a higher electrical conductivity. Sample 3 was particularly weaker than Sample 6, which may have affected the electrical conductivity. It should be noted that the PVAc did not alter the electrical properties significantly. Two different composites containing 60-wt% SWCNT and 30-wt% PH1000 with 10-wt% PVAc showed similar conductivities, ∼9×10^4^ S/m for Vinnapas 401 and ∼8.4×10^4^ S/m for Vinnapas BP600.

Figure 2B depicts thermopower values of Sample 1∼6, which were inversely proportional to the electrical conductivities. These values are lower than those of the samples containing 60 wt% SWCNT (30∼40 µV/K) [14], but higher than that of gold (1.94 µV/K at room temperature) [31]. This is another evidence that gold nanoparticles were not percolated. Sample 2 has the smallest thermopower value, which may be due to the highest electrical conductivity and shorter annealing time (mechanically weaker than Sample 5). We believe that the smaller thermopower than those of similar composites without gold can be attributed to doping.

Here, we analyzed that the influence of the gold doping on the electrical conductivity of the nanotube networks. The electrical conductivity of a composite with a high nanotube loading can be analyzed with a parallel resistance model [14], [19] and the variable range hopping model [32], [33]. The parallel resistance model describes the electrical conductivity (*σ*_c_) of a composite:
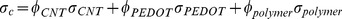
(1)where *σ_CNT_*, *σ_PEDOT_*, and *σ_polymer_* are the electrical conductivity of nanotube networks in the composite, PEDOT∶PSS, and PVAc, respectively. Also, *Φ* denotes the volume fraction of each material. Here, *σ_polymer_*≈0 because the PVAc polymer is electrically insulating (less than 10^0^ S/m) whereas the value of *σ_PEDOT_* was directly measured with 100% of PEDOT∶PSS film (∼10^2^ S/m, without dimethyl sulfoxide (DMSO) doping). Note that the electrical conductivity of PEDOT∶PSS film doped with 5 wt% of DMSO was reported as ∼10^4^ S/m [34]–[36]. In our experiments, PEDOT∶PSS was not doped with DMSO in order not to reduce thermopower of PEDOT∶PSS. The nanotubes in our composites can be assumed to be three dimensional (3D) networks and the electrical conductivity of nanotube mat, *σ_CNT_*, can be described by the 3D variable range hopping model [32], [33].
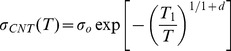
(2)
*σ_o_* is a constant, which represents the saturated electrical conductivity of nanotube networks when the temperature effect on electron carriers is negligible at infinite temperature. *T*_1_ is related to the energy barrier for electron hopping through tube-tube junctions. *T* is temperature. When *d* = 3, it represents bulk conduction of pure carbon nanotube mats [33]. The major difference between *σ_CNT_* and *σ_o_* comes from tube-tube junctions. *σ_CNT_* is for CNT networks with polymers between nanotube junctions whereas *σ_o_* is for pure tube-tube junctions without any materials in between (intrinsic properties without considering the junction effects). Therefore, it is possible to obtain the influence of the p-type doping on the electrical conductivity of the nanotube networks by comparing *σ_o_* with (indicated by Au subscript) and without (indicated by NoAu subscript) gold nanoparticles, as shown in the following equations.
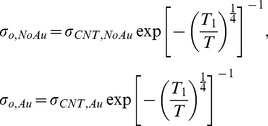
(3)The normalized factors can be obtained from *σ_o,Au_*/*σ_o,NoAu_*, and it is possible to estimate the influence of the gold doping on electrical conductivity. From Eq. (3), the normalized factor is;
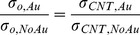
(4)The normalized factor is independent of *T*_1_ or *d*. Here, the electrical conductivity of nanotube networks, *σ_o,NoAu_* was referred from the electrical conductivity of HSWCNT mats (∼2.5×10^5^ S/m at room temperature, highest conductivity from SWCNT mats, to our best knowledge ) [37].

**Figure 3:**
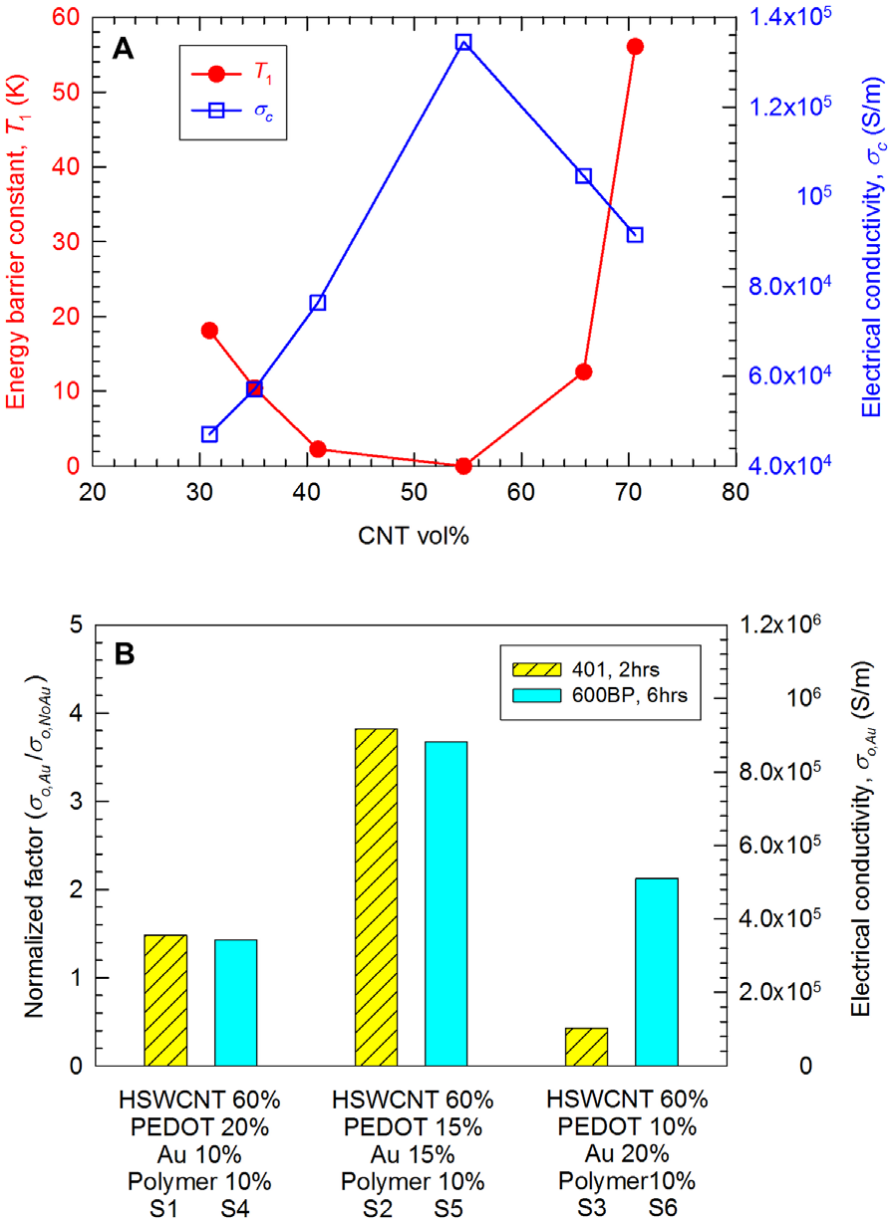
(A) The energy barrier constant, *T*_1_ in **Eq. (2)** as a function of CNT vol% (red filled circles). (B) The normalized factor (*σ_o,Au_*/*σ_o,NoAu_*) that indicates the effect of gold doping for CNT networks and electrical conductivity of gold-incorporated CNT networks (*σ_o,Au_*) for Sample 1∼6. *T*_1_ was found from the electrical conductivities (*σ*_c_) of similar composites (blue hollow squares) containing 60-wt% HSWCNT, 30-wt% PEDOT∶PSS, 10-wt% PVAc from our previous work [14]. For *σ_o,Au_* and *σ_o,NoAu_*, tube-tube junction resistances in CNT networks were not considered.

The composites with similar compositions in our previous work [14] were analyzed to obtain *T*_1_ values as a function of nanotube concentration at 300 K. Here, PEDOT∶PSS were also used to de-bundle and stabilize CNTs in water, making tube-tube junctions similar to those of the composites in this study. The composites contain 35∼75 wt% SWCNTs with PEDOT∶PSS and PVAc. The carbon nanotube wt% was converted into vol% and plotted in Figure 3A (hollow square) because the density of gold used in this work is one order higher than the polymers and CNTs. The conversion enables us to properly compare properties of the composites containing the same CNT concentrations, as described below. Then, the composite electrical conductivity (*σ*_c_) in Eq. (1), as shown in Figure 3A (filled circles), was used with *σ_CNT_* in Eq. (2) to find *T*_1_. Here, *T*_1_ at 60 vol% SWCNT concentration was obtained to be 6.06 K from the linear interpolation of 54.6 vol% (60 wt%) and 65.8 vol% (70 wt%). When we assume the tube–tube junctions are similar after the gold nanoparticle incorporation, *T*_1_ values can be used for our composites in this study. Then, we can estimate the electrical conductivity of the gold-decorated nanotubes by using Eq. (1) and (2). In other words,
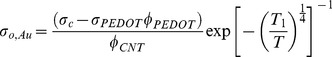
(5)
Figure 3B depicts the normalized factor (left y axis), which describes the electrical conductivity normalized by the values without gold doping (*σ_o,Au_*/*σ_o,NoAu_*). The electrical conductivity of the nanotube network with 15-vol% gold nanoparticles was increased by a factor of ∼4. However, the electrical conductivity of the composite with 20 vol% of gold nanoparticles (Sample 3) was decreased, presumably due to poor nanotube dispersions caused by a lack of the dispersant (PEDOT∶PSS) and the high concentration of gold nanoparticles.

**Figure 4:**
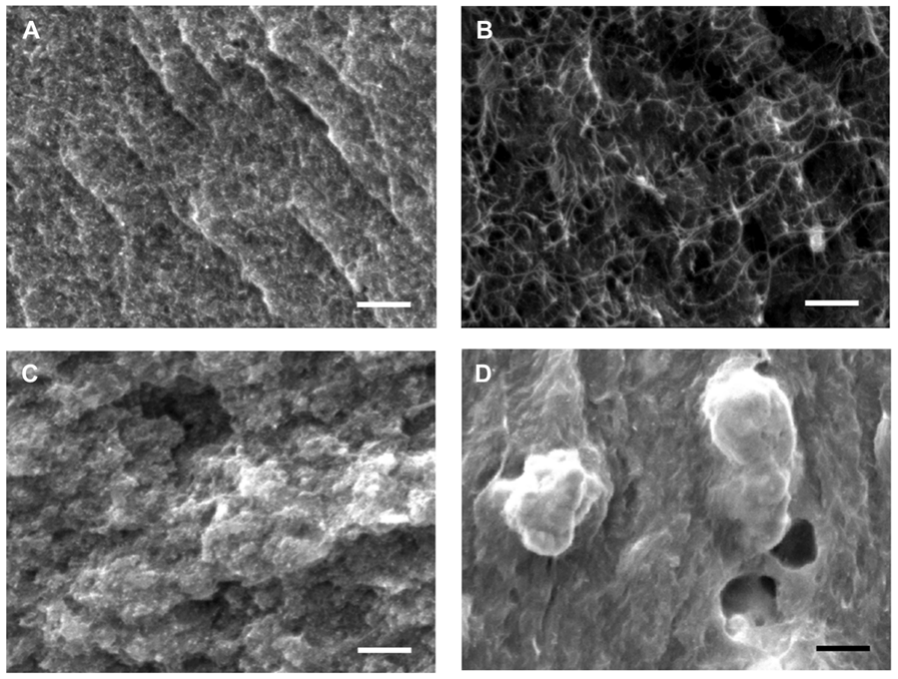
Cold-fractured cross sections of Sample 7 (A), Sample 8 (B), Sample 9 (C), and Sample 10 (D) (see Table 1). Long CSWCNTs were pulled out from the polymer whereas short MWCNTs were aggregated together in (A) and (B). Relatively rough surfaces with aggregated gold particles were observed in (C) and (D). All scale bars indicate 1 µm.

We also used different type nanotubes (MWCNT or CSWCNT) to identify the influence of the nanotubes on the electrical properties. Samples with 60-vol% CNT, 30-vol% PEDOT∶PSS, and 10-vol% polymer emulsion (Vinnapas 600BP) were prepared without gold (Sample 7 and 8). With 15-vol% gold, PEDOT∶PSS was reduced to 15 vol% (Sample 9 and 10). We found that MWCNT/CSWCNT-composites containing 15-vol% gold have higher electrical conductivities, compared to the composites containing 10 and 20-vol% gold. Sample 7 and 8 (without gold) show relatively smooth and uniform cross sections, as shown in the scanning electron micrographs of Figure 4A and 4B. More nanotubes were pulled out from the polymer with CSWCNTs (Figure 4B) than MWCNTs. This may be from inferior dispersions (i.e., more aggregations) of MWCNTs compared to SWCNTs as well as from shorter lengths of MWCNTs (1∼12 µm) [38] than SWNTs (5∼30 µm) [39]. Additionally, the number of MWCNTs is less than that of SWCNTs due to the higher density of MWCNTs. With 15-vol% gold, relatively large gold particles were observed (Figure 4C and 4D). From the energy dispersive X-ray Spectroscopy analysis, it was confirmed that the particles are comprised of gold.

**Figure 5:**
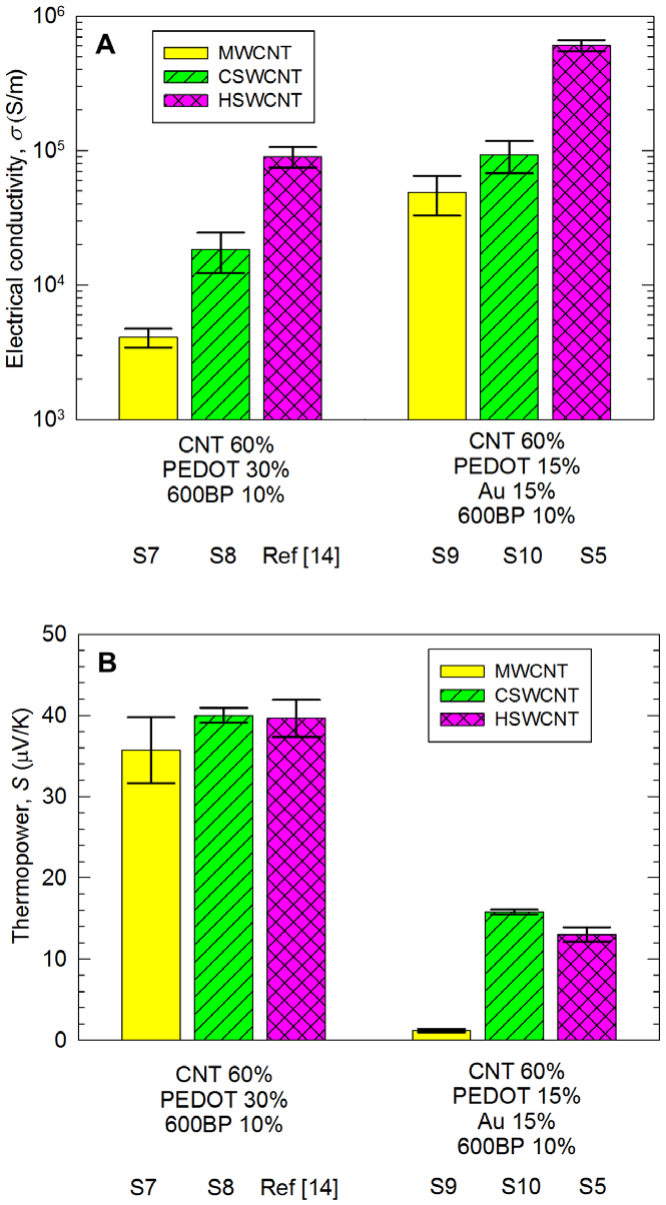
Electrical conductivity (A) and thermopower (B) of Sample 7∼10 along with those of Sample 5 and a sample in Ref. 14 for comparison. The sample in Ref. 14 contains 60-wt% (54.6 vol%) HSWCNT, 30-wt% (35.5 vol%) PEDOT∶PSS, and 10-wt% (9.9 vol%) PVAc. HSWCNT show higher conductivities than the MWCNT- and CSWCNT-filled composites, even after gold nanoparticles were incorporated.

The electrical conductivities of the composites containing three different types of nanotubes were compared in Figure 5A. The composite with MWCNT shows ∼4×10^3^ S/m, which is inferior to that of the HSWCNT sample (∼2×10^4^ S/m). In CNT networks, the electrical conductivity is often governed by tube-tube junctions [30]. When MWCNTs are used, the number of the junctions is small compared to those of SWCNT networks, diminishing electrons transport across the junctions. The large diameter of MWCNTs causes much smaller surface areas than SWCNTs. Moreover, the aggregation of MWCNTs can also be attributed to the inferior conductivity of the MWCNT sample. After replacing 15-vol% PEDOT∶PSS with 15-vol% gold in these composites, the electrical conductivities were dramatically increased to ∼7×10^4^ and ∼9×10^4^ S/m for the MWCNT/gold and CSWCNT/gold samples, respectively. Nevertheless, these values are still lower than that of the HSWCNT/gold sample. It has been reported that the intrinsic electrical conductivity of HSWCNT is higher than that of CSWCNT (approximately one order difference) [40], generally due to the higher concentration of metallic nanotubes in HSWCNT [40]. In addition, the presence of more defects such as carbonaceous particles on the surface of the CSWCNT compared to HSWCNT may cause an increase in the contact resistance between nanotubes [41]. The large difference in the electrical conductivities of the composites with CSWCNT and HSWCNT also shows that CNT networks are the electron paths rather than gold nanoparticles. Note that the electrical conductivity of bulk gold (∼4×10^7^ S/m at 300 K) [42] is at least two-order higher than that of our composites containing CSWCNT and 15-vol% gold. The thermopower values of the composites with gold nanoparticles were measured to be less than a half of those without gold, due to the large improvement in electrical conductivity by p-doping of CNT (Figure 5B).

## Conclusion

Polymer composites containing SWCNTs grown by a HipCo or CVD process or MWCNTs with PEDOT∶PSS and PVAc. The vol% of CNT and PVAc was 60 and 10, respectively. The concentration of gold nanoparticles was 0, 10, 15, and 20 vol%, and the rest was occupied by PEDOT∶PSS. Their electrical conductivities and thermopower values were measured for the composites without and with gold nanoparticles for doping CNTs. With the doping, the electrical conductivity of the composites was dramatically increased to ∼6×10^5^ S/m by replacing 15-vol% PEDOT∶PSS with gold nanoparticles. This electrical conductivity is orders of magnitude higher than those of other polymer composites containing CNTs and gold particles. Furthermore, the conductivity is ∼500% higher than those of similar composites without gold nanoparticles. We believe this is due to p-type doping caused by gold nanoparticles when they are precipitated on CNTs. A variable range hopping model with a parallel resistance model was employed to identify the change in the electrical conductivity of CNT networks in the composites. We also observed that the composites containing 20-vol% gold nanoparticles decreased the electrical conductivity due to the inferior CNT dispersions. This result indicates CNT dispersion with a proper amount of CNT dispersants is crucial to maximize electrical conductivity. Additionally, three different CNTs resulted in dissimilar electrical properties for the composites, showing that the intrinsic properties of the CNTs and dispersion are important factors. This study demonstrates nanoparticles can be used for doping CNTs to manipulate the electrical properties of CNT-filled polymer composites.

## Materials and Methods

We used three different-type CNTs: SWCNTs synthesized by a high pressure carbon monoxide (HipCo) process (HSWCNT) [43] and a chemical vapor deposition (CVD) method (CSWCNT) [39] as well as multi-wall carbon nanotubes (MWCNT) by a CVD method [38]. CNTs were added to deionized water (∼20 ml), and the solution was sonicated with an ultrasonic homogenizer (Microson XL2000, Misonix, Inc.) for 30 minutes with 50 W power. A gold ion solution was separately prepared by adding chloroauric acid (HAuCl_4_, Alfa Aesar, 99.9%) to deionized water (1∼2 ml), and then poured into the CNT solution, followed by 30 min sonication. Subsequently, an aqueous poly (3, 4-ethylenedioxythiophene) poly (styrenesulfonate) (PEDOT∶PSS, Clevios PH1000, H. C. Starck) solution was added to the mixture, followed by 15 min sonication. PEDOT∶PSS plays a role in de-bundling and dispersing CNTs in water. Finally, poly (vinyl acetate) (PVAc) emulsions were added to the mixture, followed by another 15 min sonication. Two different PVAc emulsions, Vinnapas 401 and 600BP (Wacker chemical, Co.) were used. They have different glass transition temperatures (*T_g_*): −15 and −40°C for Vinnapas 401 and 600BP, respectively. The polymer particles in the emulsion vary in size from 0.14∼3.5 µm in diameter with an average diameter of ∼650 nm. The total weight including water is typically 25 g. The aqueous mixture was then poured into a 26 cm^2^ plastic container and dried for 48 hrs under an ambient condition in a fume hood. During the drying process, the plastic container was placed on a rotating turntable (3 rpm). Sidewalls were made on the turntable in order to avoid non-uniformity of the solid contents due to air flow in the fume hood. The solid composite was then baked in a vacuum oven at 80°C for 2 or 6 hrs. The baking process helps making strong binding between nanotubes and polymers as well as removing micro voids in the composite. Finally, fully dried composites were placed in a vacuum desiccator for 24 hs in order to completely remove residual water from the composite. The thickness of the composite ranged from 27 to 40 µm.

Table 1 shows a list of samples and vol% of the materials in the composite. The actual weights of the materials are the following. For the samples containing SWCNTs with 10-, 15-, and 20-vol% gold, the weights of HAuCl_4_ respectively were 0.1094 g, 0.1263 g, and 0.1368 g; the weights of CNT respectively were 0.0256 g, 0.0197 g, and 0.0160 g; the weights of PH1000 respectively were 0.4648 g, 0.2682 g, and 0.1453 g; the weights of PVAc respectively were 0.0071 g, 0.0055 g, and 0.0044 g. The solid contents of the aqueous PH1000 [44] and PVAc [12] solutions respectively were 1.5 and 55.16 wt%. The densities of gold [42], SWCNT [45], PH1000 [44], PVAc [12] used for calculating vol% respectively were 19.3, 1.3, 1.06, and 1.19 g/cm^3^. The density of MWCNT is 2 g/cm^3^ [46], which is different from that of SWCNT. Due to the difference, the contents of the samples containing MWCNT were not the same as those of SWCNT samples. For the sample 9, the weights of MWCNT, HAuCl_4_, PH1000, and PVAc were 0.0274 g, 0.1141 g, 0.2424 g, and 0.0049 g, respectively. The samples without gold were also prepared with SWCNT and MWCNT. In sample 8, 0.0641 g of CNT, 1.7420 g of PH1000, and 0.0177 g of PVAc were mixed, whereas 0.0733 g of MWCNT, 1.2951 g of PH1000, and 0.0132 g of PVAc were used in sample 7.

Electrical conductivity was obtained by a four-point probe method (current-voltage sweeping) and thermopower was acquired by measuring temperature differences and voltages across the samples at room temperature. Details can be found from our previous work [14]. The error bars were obtained from 2–4 measurements and uncertainties associated with dimensions (length, width, and thickness of the samples) and thermocouple reading. Errors were calculated with error propagation methods [1]. For electron microscopy analysis, the composites were cold-fractured by submerging the composites in liquid nitrogen for 5 min, and then the cross section of the composites was inspected.
